# Effects of App-Based Transitional Care on the Self-Efficacy and Quality of Life of Patients With Spinal Cord Injury in China: Randomized Controlled Trial

**DOI:** 10.2196/22960

**Published:** 2021-04-01

**Authors:** Ting Liu, Sumei Xie, Yingmin Wang, Jie Tang, Xiaokuo He, Tiebin Yan, Kun Li

**Affiliations:** 1 Department of Rehabilitation Medicine The Eighth Affiliated Hospital Sun Yat-sen University Shenzhen China; 2 Department of Spinal Cord Injury Rehabilitation Guangdong Provincial Work Injury Rehabilitation Hospital Guangzhou China; 3 Department of Rehabilitation Medicine Sun Yat-sen Memorial Hospital Sun Yat-sen University Guangzhou China; 4 Department of Spinal Cord Injury Rehabilitation Sichuan Provincial Rehabilitation Hospital Chengdu China; 5 Department of Rehabilitation Medicine The Fifth Hospital of Xiamen Xiamen China; 6 School of Nursing Sun Yat-sen University Guangzhou China

**Keywords:** spinal cord injury, mobile app, transitional care, self-efficacy, quality of life

## Abstract

**Background:**

Spinal cord injury (SCI) severely impairs the physical and mental health of patients, decreasing their self-efficacy in coping with daily life and quality of life (QOL). In China, a large gap remains between the complex long-term health needs of SCI patients and the current community care system. With the prevalence of mobile terminals, the usage of mobile health apps has the potential to fill this gap by extending qualified medical resources to the families of SCI patients. Our team developed the app *Together* for the transitional care of home-dwelling SCI patients in China.

**Objective:**

This study aimed to evaluate the effects of app-based transitional care on the self-efficacy and QOL of SCI patients.

**Methods:**

Through a three-round Delphi process, an Android app was designed. Both medical staff and patients could access the app. Medical staff used it for providing remote transitional care to SCI patients. Patients used it to view transitional care time and send messages to medical staff. Thereafter, a multicenter and assessor-blinded randomized controlled trial was conducted. Participants (n=98) who had SCI and lived at home following discharge were recruited and randomly assigned to a study group (n=49) and control group (n=49) using a randomized number list in four research centers. Patients in both groups received systematic discharge education before discharge. The study group received five follow-ups conducted by trained nurses through the app, which had four core functions, namely remote assessment, health education, interdisciplinary referral, and patient interaction, at weeks 2, 4, 6, 8, and 12 following discharge. The control group received a routine telephone follow-up conducted by nurses at week 12 following discharge. The outcome measures were the Moorong Self-Efficacy Scale (MSES) and 36-item Short-Form Health Survey (SF-36) scores. Data were collected before discharge (T_0_) and at weeks 12 (T_1_) and 24 following discharge (T_2_). Differences between the groups were tested by repeated measures analysis of variance and simple effect analysis.

**Results:**

After the follow-up, the total MSES scores in the study group improved over time (T_0_=67.80, T_1_=71.90, and T_2_=76.29) and were higher than those in the control group (T_2_=64.49) at 24 weeks following discharge (simple effect analysis: *F*_1_=8.506, *P*=.004). Regarding the total SF-36 score, although it was higher in patients from the study group (T_2_=65.36) than those from the control group (T_2_=58.77) at 24 weeks following discharge, only time effects were significant (*F*_2,95_=6.671, *P*=.002) and neither the group effects nor the interaction effects influenced the change in QOL (group effects: *F*_1,96_=0.082, *P*=.78; interaction effects: *F*_2,95_=3.059, *P*=.052).

**Conclusions:**

This study confirmed that app-based transitional care improves the self-efficacy of SCI patients. Nevertheless, QOL improvement is not yet evident. Future investigations with larger sample sizes and longer observation periods are warranted to further verify the effects.

**Trial Registration:**

Chinese Clinical Trial Registry ChiCTR-IPR-17012317; http://www.chictr.org.cn/showproj.aspx?proj=19828

## Introduction

Spinal cord injury (SCI) is often a serious and life-changing disease with approximately 200,000 to 500,000 individuals newly diagnosed annually worldwide [1,2]. In China, the annual incidence of SCI is 23.7 to 60.6 persons per million [3-5]. SCI is a disastrous event for patients and their families owing to the associated lifelong disabilities and types of complications, which lead to deterioration of functioning independence [6], higher depression [7], higher readmission [8], poorer self-efficacy [1], and more problems compared with those observed in individuals without SCI. It can decrease one’s quality of life (QOL) and have high care and treatment burdens.

In China, most newly injured patients with SCI live at home after acute treatment and subacute rehabilitation [9]. They urgently require professional medical support and rehabilitation services in communities. However, there is a gap between the health needs of patients with SCI and the existing developing community care system in China. The complexity of SCI means there are more professional requirements for community medical care. The present community medical resources across China remain imbalanced. Many communities are unable to meet the needs of patients with SCI based on the competency of health professionals and basic community medical facilities [10]. Some well-equipped comprehensive hospitals and rehabilitation centers have attempted to fill the gap through transitional care, which extends professional medical care to the families of patients with SCI. In China, for patients with SCI who have just returned home from hospitals, transitional care involves continuing care services, and medical staff members provide patients with self-management knowledge and skills to help them avoid complications and unplanned readmissions, and better adapt to family and social life. The most common formats are home visits, telephone follow-ups, and outpatient services. However, some limitations continue to exist in these approaches, such as a small number of beneficiaries because of distance and insurance [11,12] and inconvenience for patients with motor disabilities receiving outpatient services [13].

With the development of the internet and prevalence of mobile terminals, such as intelligent cell phones and tablets, mobile health is playing a more important role in the Chinese medical service. A mobile health app is a form of mobile health that is installed on mobile electronic devices and has a variety of functions, including assisting in diagnosis, tele-health education, and follow-up [14]. Nowadays, apps are widely applied in the transitional care of patients with chronic diseases [15], postoperative patients [16,17], and puerperium women [18]. Numerous studies have demonstrated that app-based transitional care could enhance self-efficacy [15,18,19], prevent complications [16,17], improve QOL [20], and reduce readmissions [21] among patients.

With the development of modern medicine, one of the ultimate goals of rehabilitation for patients with SCI is to improve their QOL. QOL has been identified as one of the most common outcome indicators for patients with SCI, which could provide a comprehensive picture of the individual’s physical-psychological health and social domains [22]. Owing to the different degrees of dysfunction, patients with SCI have a marked decline in QOL compared with the general population [23]. An improvement in QOL suggests an adaptive process operating over a long period of time [24]. Interestingly, self-efficacy has been shown to be an important predictor of QOL in patients with SCI [22]. Self-efficacy is a psychological concept that describes the confidence of a person in performing specific activities and pursuing desired goals [25]. For patients with SCI, self-efficacy includes general self-efficacy and SCI-specific self-efficacy, such as self-efficacy in coping with daily life and in self-management [26]. SCI patients with low levels of self-efficacy tend to have a poor QOL [22]. In addition, low self-efficacy is strongly associated with depressive moods in patients with SCI [27]. The improvement of self-efficacy in patients with SCI is likely to promote life-long behaviors of daily life and self-regulation for health maintenance [28,29]. Therefore, improvement of the QOL and self-efficacy of patients with SCI was the focus of this study.

In view of the superiority of apps, our research team developed an app, named *Together*, specifically for the transitional care of patients with SCI. At present, our app has two user interfaces (medical staff and patients). The current language is Chinese, and the copyright is held by Sun Yat-sen University [30]. The app *Together* includes the following four core functions: remote assessment, health education, interdisciplinary referral, and patient interaction. The aim of this study was to test the effects of app-based transitional care on the self-efficacy and QOL of patients with SCI through a randomized controlled trial (RCT), thereby providing clinical evidence and guidance for the development of transitional care for patients with SCI in China.

## Methods

### Study Design and Settings

This research was a multicenter and assessor-blinded RCT (Chinese Clinical Trial Registry, ChiCTR-IPR-17012317). It was implemented in four research centers, which included one rehabilitation department in a comprehensive hospital and three rehabilitation hospitals in Guangzhou, Chengdu, and Shiyan in China. We conducted RCTs in each of the research centers over the same period.

### Development of the App

The app *Together* was developed based on the International Classification of Functioning, Disability, and Health (ICF), which offered a platform for health professionals to provide transitional care for patients with SCI at home in China. The development process of the app is scientific and rigorous. A three-round Delphi expert panel and expert consultation were performed step by step to build the framework of the app, which included the contents of remote transitional care, including SCI follow-up indicators, measurement methods, and standardized guidelines [31,32].

With regard to the construction of the SCI follow-up indicators, an ICF set for the transitional care outcome indicators of patients with SCI in China was developed through a three-round Delphi expert panel in preliminary studies [31,32]. ICF is a unified and standard terminology system for multidisciplinary use. It provides a comprehensive perspective in describing one’s functioning, and the interdisciplinary focus rendered it more suitable for multidisciplinary teamwork [33]. Each ICF category can reflect the individual’s function by using ICF qualifiers (divided into 0 to 4 levels). In our study, ICF categories were the follow-up indicators to evaluate SCI patients’ functioning. A total of 52 experts took part in the Delphi process, all of whom were nursing experts and were certified rehabilitation nurses with at least 5 years of clinical experience in nursing. Considering the feasibility of clinical practice, the outcome indicators suitable for SCI follow-up (32 for large follow-up and 12 for small follow-up) (Table 1) were selected from the ICF set above by an expert panel consisting of two rehabilitation physicians and three nursing specialists in SCI. In this study, the medical staff provided the “small follow-up” intervention at 2, 4, 6, and 8 weeks following discharge, with a total of 12 follow-up indicators. It was considered that patients needed frequent follow-ups within 3 months when they returned home, so we set less follow-up indicators to meet clinical needs. The medical staff provided the “large follow-up” intervention at 12 weeks following discharge, with a total of 32 follow-up indicators. Since patients with SCI had been discharged from the hospital for a period of time, a regular systematic follow-up for them was necessary, so we set more large follow-up indicators to comprehensively assess the patient’s current functioning. To test the validity of the SCI follow-up indicators, we used Rasch analysis [34] to examine each component of the ICF set and the overall fit to a Rasch model. The results [35] of the Rasch analysis showed good fit to the Rasch model for the different components of the ICF set in the app after modification. Both overall and single-item fits were satisfactory. These results indicated the suitability of our selected ICF set as follow-up indicators to evaluate the functioning of patients with SCI.

With regard to the construction of measurement methods and standardized guidelines, for each follow-up indicator, the operational measurement method was set based on literature review and existing measurements by expert consultation [36]. We transformed patient information into ICF qualifiers according to different criteria, such as percentages, frequency, and medical staff assessment results. For example, for *b4200 Increased blood pressure* and *b4201 Decreased blood pressure*, we transformed patient information into ICF qualifiers according to frequency. Stable blood pressure over the past month was rated as 0, whereas high/low blood pressure almost every day received a rating of 4. Our app can automatically transform routine clinical assessments into the unified ICF qualifiers (“0,” no problem; “1,” mild problem; “2,” moderate problem; “3,” serious problem; “4,” complete problem; and “9,” not applicable) for better understanding among the multidisciplinary team. Moreover, a standardized health education program for each follow-up indicator was developed by expert consultation based on the Knowledge-Attitude-Practice theory [37].

Based on previous work, software engineers developed the mobile app using the Java language according to the requirements set by the researchers [31]. Eventually, an Android version of the app was designed. Visitors to the app could be medical staff or patients, but they were required to apply for registration with their real identity first. Thereafter, the researchers verified applicants’ information before they could log in. Unregistered users could not access the app even if they had downloaded it. In addition, medical staff and patients had different access interfaces after logging in. Medical staff entered a follow-up system and used it to provide transitional care for patients with SCI. The patient interface could be only used to view follow-up time and send messages to the medical staff. For each follow-up, medical staff needed to record the patient’s follow-up outcomes in the app. The collected information was stored in the cloud, which was actually a cloud computing server like Google cloud for data storage with good security. The security measures of our app were as follows: (1) user login required a password for secure login; (2) users’ sensitive information was encrypted and saved to prevent data leakage; and (3) data were regularly saved to the cloud to prevent loss.

**Table 1:** Follow-up outcome indicators of transitional care for patients with spinal cord injury.

Classification	Variable	Small follow-up	Large follow-up^a^
ICF^b^ category	Body function	*b280 Sense of pain* *b415 Blood vessel functions* *b435 Immunological system functions* *b440 Functions of the respiratory system* *b4200 Increased blood pressure* *b4201 Decreased blood pressure* *b530 Weight maintenance functions*	*b134 Sleep functions* *b152 Emotional functions* *b280 Sense of pain* *b415 Blood vessel functions* *b4200 Increased blood pressure* *b4201 Decreased blood pressure* *b435 Immunological system functions* *b440 Functions of the respiratory system* *b530 Weight maintenance functions* *b640 Sexual functions* *b660 Procreation functions* *b710 Mobility of joint functions* *b730 Muscle power functions* *b735 Muscle tone functions*
ICF category	Body structure	*s810 Structure of areas of skin*	*s810 Structure of areas of skin*
ICF category	Activities and participation	*d5300 Regulating urination* *d5301 Regulating defecation* *d410 Changing basic body position* *d420 Transferring oneself*	*d410 Changing basic body position* *d420 Transferring oneself* *d450 Walking* *d465 Moving around using equipment* *d510 Washing oneself* *d520 Caring for body parts* *d5300 Regulating urination* *d5301 Regulating defecation* *d540 Dressing* *d550 Eating* *d560 Drinking* *d9205 Socializing* *d760 Family relationships*
ICF category	Contextual factors^c^	N/A^d^	*e1201 Assistive products and technology for personal indoor and outdoor mobility and transportation* *e155 Design, construction, and building products and technology of buildings for private use* *Acceptance of life in a wheelchair/in bed* *Coping with everyday life* *Adjustment to new body image* *Knowledge about spinal cord injury*
Concepts not covered in the ICF^e^	N/A	*Ability of the caregiver*	*Ability of the caregiver*

^a^The large follow-up indicators include all small follow-up indicators.

^b^ICF: International Classification of Functioning, Disability, and Health.

^c^The contextual factors consisted of environmental factors and personal factors. Considering medical staff’s heavy workload, it is not realistic to evaluate four components of ICF at each follow-up. The small follow-up was conducted within 3 months following discharge. During this time, we paid more attention to the health conditions of patients. Thus, the contextual factors were not included in the small follow-up.

^d^N/A: not applicable.

^e^Through the Delphi process, we exacted one concept not covered in the ICF (ability of the caregiver). This reflected the views of Chinese experts.

### Participants

Participants were recruited from May 2018 to December 2019. The inclusion criteria were as follows: (1) diagnosis of complete or incomplete SCI according to the International Standards for the Neurological Classification of SCI of the American Spinal Injury Association, 2016 [38], with confirmation by computed tomography/magnetic resonance imaging (either traumatic or nontraumatic); (2) onset of SCI within the past 2 years; (3) being alert and conscious, and capable of daily verbal communication; (4) age >18 years; (5) living at home following discharge; (6) availability of mobile terminals and internet access at home; and (7) familiarity with the usage of mobile terminals. The exclusion criteria were as follows: (1) congenital spinal cord disease; (2) severe cardiovascular, brain, pulmonary, liver, or renal complications; and (3) admission to other medical institutions following discharge.

Randomization was performed using the software Research Randomizer [39]. This software generated a random number list for each center with two numbers in the list (“1” and “2”), which referred to the study group and control group, respectively. According to the discharge sequence and corresponding randomized number, the recruited patients were randomly assigned to the study group and control group. The unused numbers were discarded after completion of the study.

The sample size was estimated based on the primary outcome measure (Moorong Self-Efficacy Scale [MSES] score) between the groups. In the study conducted by Martin et al [40], the mean difference in the MSES score at week 12 was normally distributed, with a mean of 1.95 and a standard deviation (SD) of 2.77. A total of 84 patients (42 patients in each group) were required for the study, with a two-tailed α of .05 and a power of 0.80. We planned to recruit 98 patients in total to allow for a 15% dropout rate.

### Pilot Study

Prior to the RCT, a pilot study was conducted. First, for testing the functions of the app, we selected a multidisciplinary team that included two nurses, a rehabilitation physician, a physical therapist, and an occupational therapist, and 20 patients with SCI who met the inclusion criteria to download and use the app at one research center in Guangzhou. Owing to limited time, we only tested the first small follow-up. Both medical staff and patients were asked about the app’s user experience through a face-to-face interview. From the interview, we learned that medical staff generally believed that the app can help them to carry out follow-up visits in terms of comprehensive content, a user-friendly interface, and simple operation, and patients found it easy to contact their health care providers through the app. Subsequently, the researchers and software engineers optimized the app according to the suggestions. Second, for testing the validity and reliability of the outcome measures, 20 patients were asked to fill out the MSES and QOL scale at baseline.

### Interventions

Both the study and control groups received routine systematic discharge education provided by the medical team in the research centers before discharge, including information on medication, bladder management, bowel management, respiratory training, activities of daily living training, and prevention of complications (pressure ulcers, infections, falls, autonomic dysfunction, orthostatic hypotension, deep vein thrombosis, etc).

#### Study Group

A multidisciplinary team, including one follow-up nurse, one rehabilitation physician, one physical therapist, and one occupational therapist, managed the app-based transitional care in each center. The nurse was in charge of the team and responsible for the app-based transitional care. All follow-up nurses were required to have over 3 years of working experience in caring for patients with SCI.

The follow-up nurses provided information on the installation and usage of the app to the patients and their main caregivers in the study group prior to discharge. Five follow-ups based on the app were conducted for the study group by the multidisciplinary team at 2, 4, 6, 8, and 12 weeks following discharge. The timing and frequency of postdischarge interventions were set according to previous studies and the feasibility of this study. A longitudinal study suggested that the QOL of patients with SCI at 3 months following discharge was an important predictor of QOL at 15 months [41]. One telephone counselling program that delivered seven tele-counseling sessions over a 12-week period was effective in managing psychological outcomes [42]. Therefore, the researchers focused on patients with SCI who were discharged from the rehabilitation center within 3 months and developed the transitional care plan with five follow-ups based on the app within 3 months following discharge. The app would automatically remind the follow-up nurse of the patients that had to be contacted within 1 week prior to the end of the follow-up. The app-based transitional care intervention provided by the multidisciplinary team for patients with SCI included the following four parts: (1) remote health assessment, (2) health education, (3) interdisciplinary referral, and (4) patient interaction.

##### Remote Health Assessment

**Figure 1:**
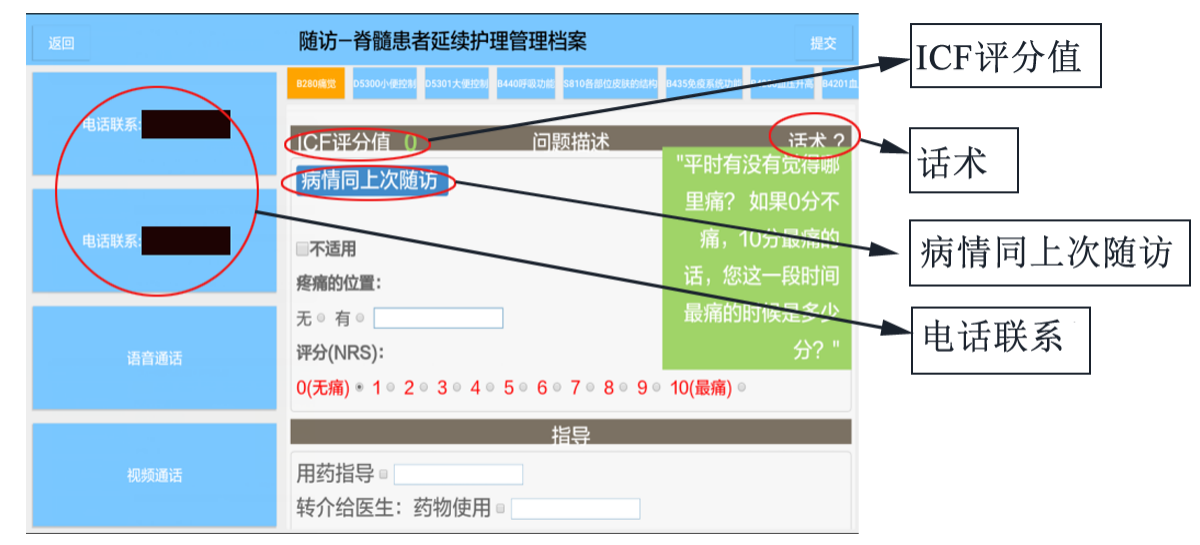
Remote assessment interface for the category of "b280 Sense of pain." ICF评分值 means “ICF qualifiers.” After clicking, ICF qualifiers will be shown. 话术 means “verbal prompt.” After clicking, preset instructions will be shown. 病情同上次随访 means “The current condition is the same as the last follow-up.” 电话联系 means “Telephone.” The phone number of the patient is shown here. After clicking, the follow-up nurse can contact the patient by telephone. ICF: International Classification of Functioning, Disability, and Health.

In the follow-up system of the medical staff interface, ICF categories were displayed as follow-up indicators. The nurse could remotely evaluate patients’ functioning by questioning patients via telephone according to preset instructions on the app. A verbal prompt was provided in the medical staff interface of the app to guide them in implementing a standard assessment, and the app automatically transformed the original clinical assessment results to the ICF qualifiers according to preset operational measuring guidelines. For example, for *b280 Sense of pain* (Figure 1), the verbal prompt was “If 0 is not painful and 10 is the most painful, how serious is your pain?” After the nurse selected the appropriate number from 0 to 10 according to the patient’s answer, the app automatically transformed the assessment results to ICF qualifiers. If the patient’s condition did not change greatly from the last follow-up, the nurse could click on “The current condition is the same as the last follow-up.” Subsequently, the system could achieve this automatically. The setting can automatically synchronize the results of the last follow-up, which not only facilitates the medical staff to obtain the patients’ previous functional status, but also saves their time.

##### Health Education

**Figure 2:**
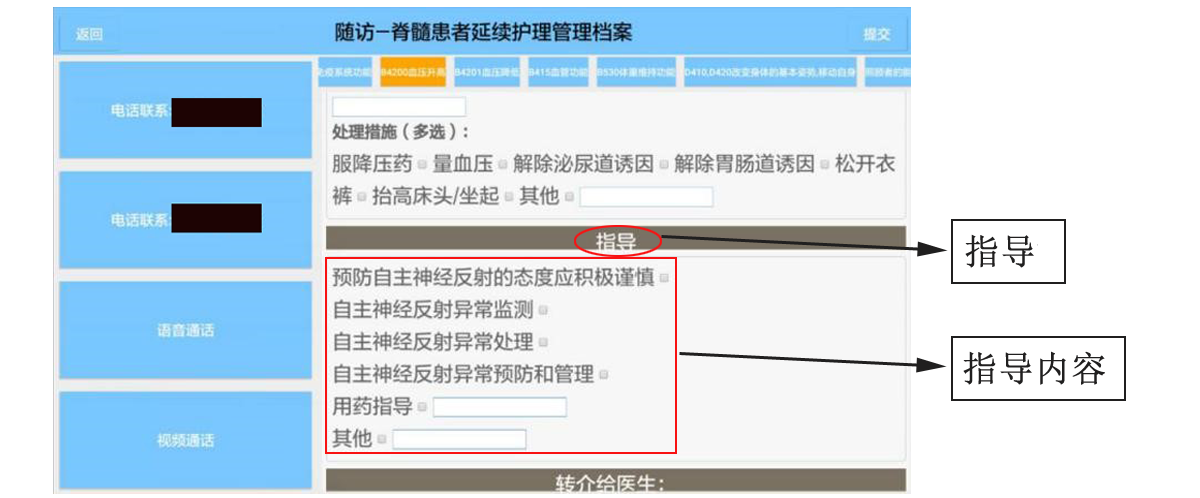
Health education interface for the category of "b4200 Increased blood pressure." 指导 means “Health education.” 指导内容 means “Health education contents.” The health education contents for the ICF category "b4200 Increased blood pressure" are as follows: (1) the attitude toward preventing AD is positive and cautious; (2) instructing patients in the monitoring of AD; (3) instructing patients in the treatment of AD; (4) instructing patients in the prevention and management of AD; (5) medication guidance; and (6) others. AD: autonomic dysreflexia; ICF: International Classification of Functioning, Disability, and Health.

The app provided a standard health education framework on each ICF category based on the Knowledge-Attitude-Practice theory. The health education framework of each ICF category was only visualized in the medical staff interface of the app. The follow-up nurses combined their own clinical experience and knowledge to provide health education for patients according to the prompted and standard health education framework in the app. Figure 2 shows the health education framework for the ICF category of *b4200 Increased blood pressure* in the app. The education program included emphasizing the importance of preventing autonomic dysreflexia (AD), checking the monitoring records regarding AD, reviewing emergency management for the occurrence of AD, assessing the prevention and management of AD, providing medication guidance, etc.

##### Interdisciplinary Referral

**Figure 3:**
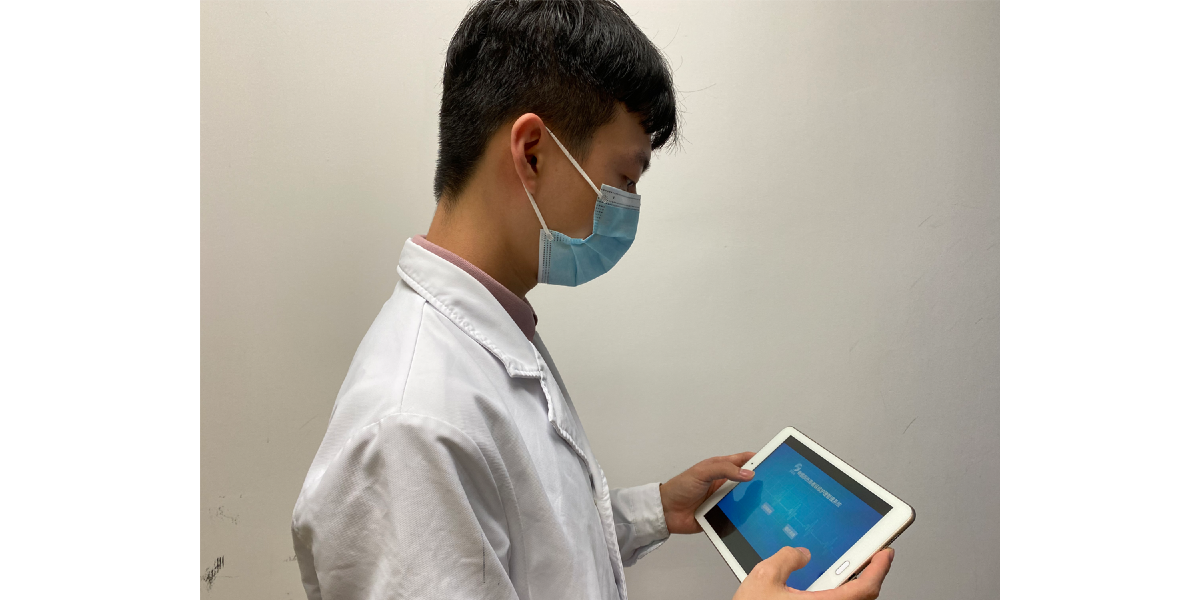
An occupational therapist using the app.

The referral function is a closed loop processing mode. When the follow-up nurses encountered problems that did not belong to the SCI nursing specialty, such as drug use and home modifications, referral messages were sent by the nurses to the corresponding members of the multidisciplinary team (eg, rehabilitation physician, physical therapist, or occupational therapist) through the app. The team members were required to log in to the app within 3 days and handle the referral problems by contacting the patients. In the end, the physician or therapist needed to fill in the referral record in the app. If the specialist was unable to contact the patient within 3 days, the referral record was not allowed to be filled in anymore and the patient was forced to withdraw from our study. Figure 3 shows an occupational therapist using the app.

##### Patient Interaction

Medical staff could communicate with patients via telephone and the messaging function of the app. In turn, patients could contact medical staff by sending messages. Before every follow-up or contacting patients, medical staff read the messages received. In addition, nurses dealt with the messages from patients every Monday. If they encountered problems that they could not handle, they needed to send referral messages to other specialists. Relevant specialists were required to log in to the app to contact patients within 3 days.

#### Control Group

No transitional care service was provided to the control group in the 3 months following discharge. At 12 weeks following discharge, the patients in the control group received a routine telephone follow-up conducted by nurses to mainly assess the functional level and complications of the patients and the disease management knowledge and skills of the patients and their caregivers. Corresponding health education was also included.

### Outcome Measures

Demographic and disease-related data were collected before discharge. All patients were assessed using the MSES and 36-item Short-Form Health Survey (SF-36) at baseline (prior to discharge; T_0_), 12 weeks following discharge (T_1_), and 24 weeks following discharge (T_2_).

#### Demographic Disease Characteristics

The demographic disease inquiry consisted of two parts, namely demographic information and disease information. Patients were required to fill out the demographic information (name, gender, age, education background, marital status, insurance, occupation status, per capita income, main caregivers, etc), and nurses filled out the disease information (diagnosis, injury level, injury severity, causes, disease duration, etc) according to medical records.

#### MSES

MSES is a 16-item self-report scale developed by Middleton that measures one’s ability to control behavior and outcomes, specifically among patients with SCI [43]. It consists of three-factor structures (social function self-efficacy, general self-efficacy, and personal function self-efficacy), and utilizes a 7-point Likert scale ranging from 1 (very uncertain) to 7 (very certain). Higher scores indicate better self-efficacy of patients. In a previous study, it exhibited adequate reliability (Cronbach α of .94) and validity (content validity index [CVI] of 0.91) [44,45]. With the permission of the original authors, we developed a Chinese version of the MSES through forward and backward translation. In the study, the scale CVI was 0.99 (evaluated by eight clinical nursing specialists) and the item CVI ranged from 0.88 to 1.00. In addition, the Cronbach α was .91 in a pilot test.

#### SF-36

SF-36 is commonly used to measure the health-related QOL of patients with SCI [46]. It contains 36 items that measure perceived health in eight domains (physical functioning, role-physical, bodily pain, general health, vitality, social functioning, role-emotional, and mental health), with higher scores (range 0-100) reflecting better perceived health. The first four domains form the physical component summary (PCS), and the last four domains form the mental component summary (MCS). The Chinese version of the SF-36 was translated in 1998 [47]. When applied in patients with SCI, the Cronbach α values of the domains of the SF-36 ranged from .76 to .90 [48].

### Procedure

First, face-to-face training for the follow-up team members of each research center was conducted simultaneously. The training lasted 4 hours and consisted of an introductory lecture on the study and app, as well as a workshop on the operation of the app. All members participated in a qualification test after training, with a minimum requirement score of ≥80/100.

Subsequently, the RCT was performed after the training. Eligible patients were recruited prior to discharge and randomly assigned to the study or control group according to their sequence of discharge. Both groups received routine systematic discharge education, and the patients in the study group received guidance on the usage of the app. The patients or medical staff downloaded the app by scanning a QR code. A verification system was set in the app, and only patients in the study group could pass the identity verification and use the patient interface of the app. The study group underwent five app-based follow-ups (at 2, 4, 6, 8, and 12 weeks following discharge) performed by the multidisciplinary team, while the control group underwent one routine telephone follow-up conducted by nurses. The outcome measures were collected by researchers at T_0_, T_1_, and T_2_ by sending online survey links to the mobile phones of patients. After completing the data collection, patients received a US $3 reward online.

### Ethical Considerations

The study protocol was approved by the ethics committee of Sun Yat-sen University (2017ZSLYEC-062). All patients were informed of the research process, the aims, their privacy protection, and their rights. All patients were required to sign the informed consent form. For patients who could not sign the consent form owing to disabilities, consent was considered if the patient verbally agreed in the presence of a witness.

### Statistical Analysis

SPSS version 25.0 software (IBM Corp) was used for data analysis. The frequency, percentage, mean, SD, median, and IQR were used to describe the demographic and disease-related data of the patients in the study group and those in the control group. Mean (SD) or median (IQR) was used to describe the scores of the MSES and SF-36. At the baseline assessment, the chi-squared test, *t* test, or Wilcoxon rank-sum test was used for balance testing of all variables, including demographic disease characteristics and outcome measures, between the study and control groups. The normality of the outcome indicators was tested and Box-Cox transformation was used to transform nonnormally distributed data into normally distributed data. Repeated measures analysis of variance was used to analyze the main effects of time, group, and time*group interaction on the MSES and SF-36 scores. In the presence of an interaction effect, simple effect analysis was further performed. A *P* value <.05 was considered to indicate a statistically significant difference.

## Results

### Response to Follow-Up

**Figure 4:**
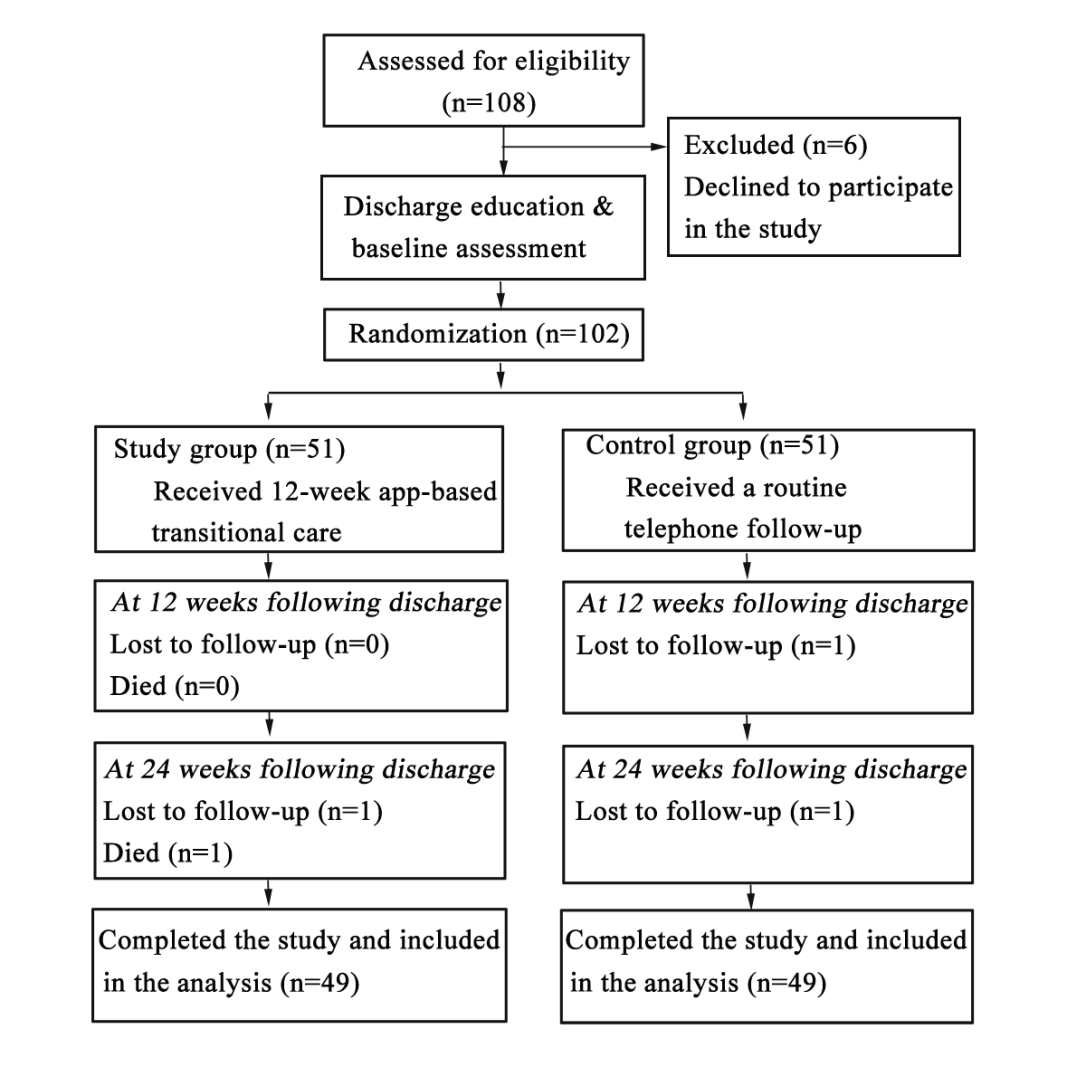
The CONSORT flow diagram of this study.

Figure 4 shows the CONSORT (Consolidated Standards of Reporting Trials) flow diagram of this study. Patients with SCI (n=108) were assessed for eligibility, and 102 patients meeting the inclusion criteria were randomly allocated to the study group and the control group. Eventually, only 98 patients completed the study (49 patients per group). In the study group, two patients were lost to follow-up at week 24. The reasons were suicide and disconnection in the two patients. In the control group, two patients were lost to follow-up (owing to disconnection) at weeks 12 and 24. The overall effective follow-up rate in this study was 96.1% (98/102), and the attrition rate was 3.9% (4/102).

### Balance Test

#### Between the Group Within Guangzhou and the Group Outside Guangzhou

There were two research centers within Guangzhou and two outside Guangzhou (in Shiyan and Chengdu). Because our study researcher team was in Guangzhou, it was easier to control the study quality of research centers within Guangzhou. Therefore, we divided the four centers into two groups according to the region (the group within Guangzhou and the group outside Guangzhou). As shown in Table 2, there were no statistically significant differences in the demographic and disease-related data and the baseline scores of self-efficacy and QOL among patients between the group within Guangzhou and the group outside Guangzhou. Thus, we regarded the patients in the four research centers as a whole and carried out repeated measures analysis of variance to analyze the outcome indicators between the study group and control group.

**Table 2:** Balance test between the group within Guangzhou and the group outside Guangzhou (T_0_).

Variable		Group within Guangzhou (n=34)	Group outside Guangzhou (n=64)	Total (n=98)	*t*/χ^2^/Z	*P* value
Age (years), mean (SD)		41.62 (13.51)	41.77 (11.46)	41.71 (12.14)	t_96_=−0.06	.96^a^
**Sex, n (%)**					χ^2^_1_=0.25	.62^b^
	Male	29 (85)	52 (81)	81 (83)		
	Female	5 (15)	12 (19)	17 (17)		
**Education level, n (%)**					χ^2^_3_=1.40	.71^b^
	Primary school	9 (27)	19 (30)	28 (29)		
	Junior high school	13 (38)	27 (42)	40 (41)		
	High school	8 (24)	9 (14)	17 (17)		
	College or above	4 (12)	9 (14)	13 (13)		
**Marital status, n (%)**					χ^2^_1_=0.79	.37^b^
	Unmarried/divorced	4 (12)	12 (19)	16 (16)		
	Married	30 (88)	52 (81)	82 (84)		
**Income (RMB/person/month), n (%)**					χ^2^_2_=5.63	.06^b^
	<3000	18 (53)	47 (73)	65 (66)		
	3000-4999	9 (27)	13 (20)	22 (22)		
	≥5000	7 (21)	4 (6)	11 (11)		
**Occupation, n (%)**					N/A^c^	.57^d^
	Employed	2 (6)	3 (5)	5 (5)		
	Unemployed	32 (94)	61 (95)	93 (95)		
**Disease duration (months), n (%)**					χ^2^_1_=0.52	.47^b^
	0-12	29 (85)	59 (92)	88 (90)		
	13-22	5 (15)	5 (8)	10 (10)		
**Etiology, n (%)**					N/A	>.99^d^
	Traumatic	31 (91)	58 (91)	89 (91)		
	Nontraumatic	3 (9)	6 (9)	9 (9)		
**Injury severity, n (%)**					χ^2^_1_=0.08	.78^b^
	Complete SCI^e^	16 (47)	32 (50)	48 (49)		
	Incomplete SCI	18 (53)	32 (50)	50 (51)		
**Injury level, n (%)**					χ^2^_2_=0.17	.92^b^
	Cervical	11 (32)	18 (28)	29 (30)		
	Thoracic	15 (44)	31 (48)	46 (47)		
	Lumbar/sacral/cauda equina	8 (24)	15 (23)	23 (24)		
**MSES^f^** ** score, mean (SD)**		67.80 (20.68)	74.06 (21.64)	70.93 (21.29)	t_96_=−1.47	.15^a^
	Social function self-efficacy	23.55 (6.73)	25.14 (6.75)	24.35 (6.75)	t_96_=−1.17	.25^a^
	General self-efficacy	16.25 (5.75)	17.84 (6.21)	17.04 (6.00)	t_96_=−1.32	.19^a^
	Person function self-efficacy	28.00 (9.79)	31.08 (11.01)	29.54 (10.48)	t_96_=−1.46	.15^a^
**SF-36^g^** ** score, median (IQR)**		58.69 (52.29-81.16)	57.69 (49.89-68.48)	58.16 (50.53-71.30)	*Z*=−1.23	.22^h^
	PCS^i^	20.28 (17.95-29.91)	18.06 (13.41-21.63)	19.17 (14.64-26.40)	*Z*=−0.90	.37^h^
	MCS^j^	36.78 (30.95-52.76)	40.65 (30.69-45.63)	38.63 (30.95-46.04)	*Z*=−0.01	.99^h^

^a^*P* value of the independent sample *t* test between the study and control groups.

^b^*P* value of the chi-square test between the study and control groups.

^c^N/A: not applicable.

^d^*P* value of the Fisher exact test between the study and control groups.

^e^SCI: spinal cord injury.

^f^MSES: Moorong Self-Efficacy Scale.

^g^SF-36: 36-item Short-Form Health Survey.

^h^*P* value of the Wilcoxon rank-sum test between the study and control groups.

^i^PCS: physical component summary.

^j^MCS: mental component summary.

#### Between the Study and Control Groups

There was no significant difference in baseline data between the study and control groups (Table 3). On average, the majority of patients were male (81/98, 83%) with a mean age of 41.71 (SD 12.14) years (range 18-65 years). Most patients had an education level of junior high school or below (68/98, 69%). Almost 95% (93/98) of patients were unemployed. Prior to the injury, the majority were workers and farmers (workers: 55/98, 56%; farmers: 19/98, 19%).

The disease duration in most patients was less than 1 year (88/98, 90%) (range 1-22 months). Traumatic SCI accounted for 91% (89/98) of cases. Among them, the top three causes of the disease were falling from a height, traffic accidents, and being struck by objects (42/98, 43%; 31/98, 32%; and 13/98, 13%; respectively). The numbers of patients with complete and incomplete SCI were almost equal. Thoracic SCI was the most common injury (46/98, 47%).

**Table 3:** Balance test between the study and control groups.

Variable			Study group (n=49)		Control group (n=49)		Total (n=98)		*t*/χ^2^/Z		*P* value
Age (years), mean (SD)			40.37 (12.18)		43.06 (12.06)		41.71 (12.14)		t_96_=−1.10		.27 ^a^
**Sex, n (%)**									χ^2^_1_=0.07		.79^b^
	Male	41 (84)		40 (82)		81 (83)					
	Female	8 (16)		9 (18)		17 (17)					
**Education level, n (%)**									χ^2^_3_=4.15		.25^b^
	Primary school	16 (33)		12 (25)		28 (29)					
	Junior high school	20 (41)		20 (41)		40 (41)					
	High school	5 (10)		12 (25)		15 (17)					
	College or above	8 (16)		5 (10)		10 (11)					
**Marital status, n (%)**									χ^2^_1_=0.00		>.99^b^
	Unmarried/divorced	8 (16)		8 (16)		16 (16)					
	Married	41 (84)		41 (84)		82 (84)					
**Income (RMB/person/month), n (%)**									χ^2^_2_=1.39		.50^b^
	<3000	35 (71)		30 (61)		65 (66)					
	3000-4999	10 (20)		12 (25)		22 (22)					
	≥5000	4 (8)		7 (14)		11 (11)					
**Occupation, n (%)**									N/A^c^		>.99^d^
	Employed	3 (6)		2 (4)		5 (5)					
	Unemployed	46 (94)		47 (96)		93 (95)					
**Disease duration (months), n (%)**									χ^2^_1_=0.45		.51^b^
	0-12	45 (92)		43 (88)		88 (90)					
	13-22	4 (8)		6 (12)		10 (10)					
**Etiology, n (%)**									N/A		>.99^d^
	Traumatic	45 (92)		44 (90)		89 (91)					
	Nontraumatic	4 (8)		5 (10)		9 (9)					
**Injury severity, n (%)**									χ^2^_1_=0.16		.69^b^
	Complete SCI^e^	25 (51)		23 (47)		48 (49)					
	Incomplete SCI	24 (49)		26 (53)		50 (51)					
**Injury level, n (%)**									χ^2^_2_=0.17		.92^b^
	Cervical	14 (29)		15 (31)		29 (30)					
	Thoracic	24 (49)		22 (45)		46 (47)					
	Lumbar/sacral/cauda equina	11 (22)		12 (25)		23 (24)					
**MSES^f^** ** score, mean (SD)**			67.80 (20.68)		74.06 (21.64)		70.93 (21.29)		t_96_=−1.47		.15^a^
	Social function self-efficacy	23.55 (6.73)		25.14 (6.75)		24.35 (6.75)		t_96_=−1.17		.25^a^	
	General self-efficacy	16.25 (5.75)		17.84 (6.21)		17.04 (6.00)		t_96_=−1.32		.19^a^	
	Person function self-efficacy	28.00 (9.79)		31.08 (11.01)		29.54 (10.48)		t_96_=−1.46		.15^a^	
**SF-36^g^** ** score, median (IQR)**			57.55 (49.71-67.07)		59.50 (51.44-71.69)		58.16 (50.53-71.30)		*Z*=−0.71		.48^h^
	PCS^i^	19.38 (14.14-25.11)		18.70 (14.94-27.20)		19.17 (14.64-26.40)		*Z*=−0.05		.96^h^	
	MCS^j^	38.27 (31.40-45.02)		38.85 (30.02-48.08)		38.63 (30.95-46.04)		*Z*=−0.49		.62^h^	

^a^*P* value of the independent sample *t* test between the study and control groups.

^b^*P* value of the chi-square test between the study and control groups.

^c^N/A: not applicable.

^d^*P* value of the Fisher exact test between the study and control groups.

^e^SCI: spinal cord injury.

^f^MSES: Moorong Self-Efficacy Scale.

^g^SF-36: 36-item Short-Form Health Survey.

^h^*P* value of the Wilcoxon rank-sum test between the study and control groups.

^i^PCS: physical component summary.

^j^MCS: mental component summary.

### MSES in the Study and Control Groups

**Figure 5:**
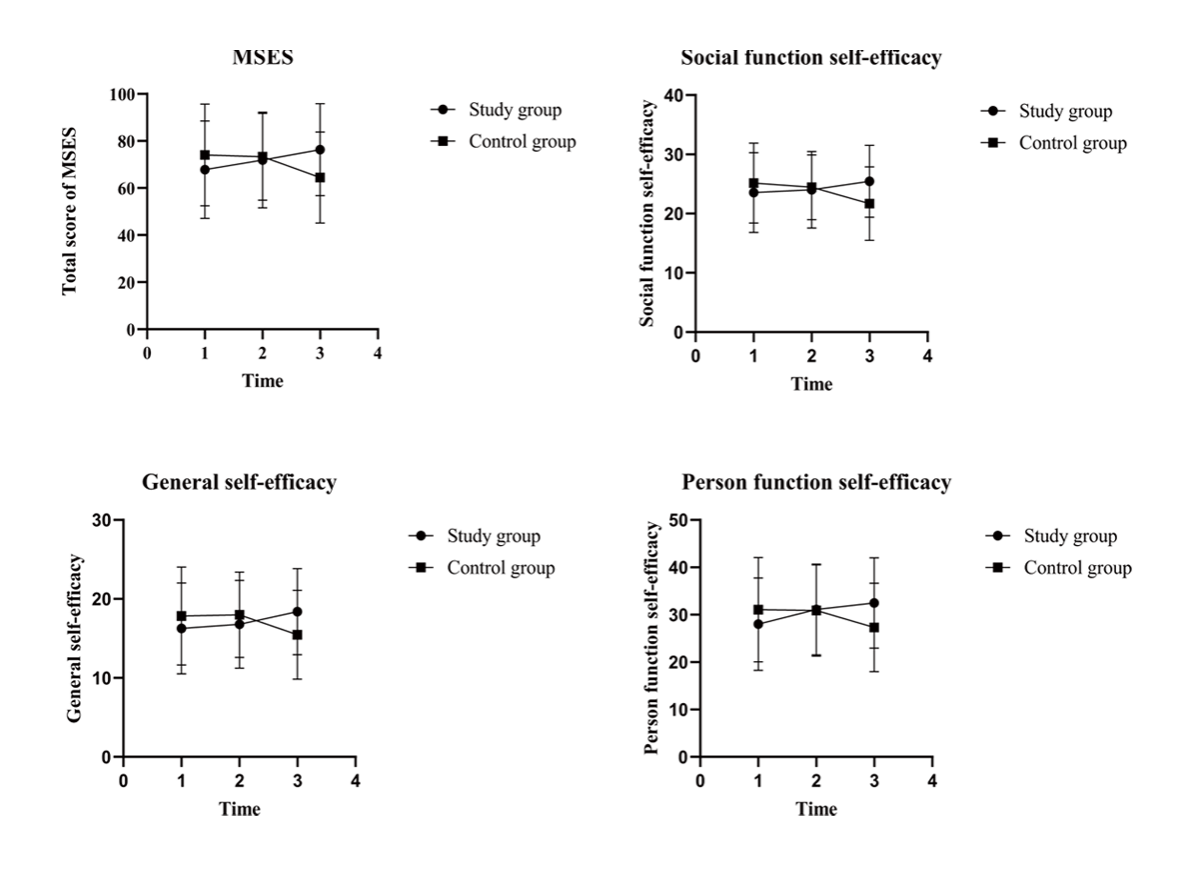
Trend charts of total Moorong Self-efficacy Scale (MSES) scores and scores of three-factor structures. Time 1: prior to discharge (baseline); time 2: at 12 weeks following discharge; time 3: at 24 weeks following discharge.

The total scores and three-factor structure scores of the MSES for the study and control groups at T_0_, T_1_, and T_2_ are shown in Table 4. There were no differences between the groups in time effects and group effects. However, differences in the interaction effects were statistically significant (total scores: *F*_2,95_=20.389, *P*<.001; social function self-efficacy: *F*_2,95_=13.445, *P*<.001; general self-efficacy: *F*_2,95_=16.063, *P*<.001; person function self-efficacy: *F*_2,95_=13.604, *P*<.001). Simple effect analysis was applied to further compare the scores of the study and control groups (Table 5). The total scores and three-factor structure scores at T_2_ were significantly higher in the study group than in the control group (total scores: *F*_1_=8.506, *P*=.004; social function self-efficacy: *F*_1_=8.698, *P*=.003; general self-efficacy: *F*_1_=6.684, *P*=.01; person function self-efficacy: *F*_1_=6.684, *P*=.01). Trend charts (Figure 5) showed that the total score and scores of the three-factor structures in the study group trended upward over time. In contrast, the scores in the control group showed a decreasing trend over time.

**Table 4:** Repeated measures analysis of variance for the Moorong Self-Efficacy Scale and the 36-item Short-Form Health Survey between the study group (n=49) and control group (n=49).

Outcome		T_0_^a^	T_1_^b^	T_2_^c^	Time effect			Group effect			Interaction effect	
					*F*_2,95_	*P* value	*F*_1,96_		*P* value	*F*_2,95_		*P* value
**MSES^d^ score^e^, mean (SD)**					1.556	.22	0.144		.71	20.389		<.001
	Study group	67.80 (20.68)	71.90 (20.36)	76.29 (19.50)								
	Control group	74.06 (21.64)	73.33 (18.46)	64.49 (19.33)								
**Social function self-efficacy^e^, mean (SD)**					1.234	.30	0.275		.60	13.445		<.001
	Study group	23.55 (6.73)	24.00 (6.49)	25.45 (6.06)								
	Control group	25.14 (6.75)	24.45 (5.46)	21.69 (6.21)								
**General self-efficacy^e^, mean (SD)**					0.607	.55	0.001		.97	16.063		<.001
	Study group	16.25 (5.75)	16.78 (5.56)	18.38 (5.46)								
	Control group	17.84 (6.21)	17.98 (5.42)	15.47 (5.64)								
**Person function self-efficacy^e^, mean (SD)**					1.745	.18	0.193		.66	13.604		<.001
	Study group	28.00 (9.79)	31.12 (9.62)	32.47 (9.57)								
	Control group	31.08 (11.01)	30.90 (9.65)	27.33 (9.36)								
**SF-36^f^ score^g^, median (IQR)**					6.671	.002	0.082		.78	3.059		.05
	Study group	57.55 (49.71-67.07)	61.06 (52.23-74.67)	65.36 (59.98-78.54)								
	Control group	59.50 (51.44-71.69)	58.83 (53.24-71.74)	58.77 (53.37-79.19)								
**PCS^h^ score^g^, median (IQR)**					24.516	<.001	0.459		.50	2.551		.08
	Study group	19.38 (14.14-25.11)	27.20 (19.37-33.50)	23.92 (20.32-32.80)								
	Control group	18.70 (14.94-27.20)	25.66 (20.13-31.06)	20.80 (15.11-28.12)								
**MCS^i^ score^g^, median (IQR)**					2.039	.14	0.086		.77	0.421		.66
	Study group	38.27 (31.40-45.02)	30.60 (26.17-49.58)	40.01 (32.03-49.59)								
	Control group	38.85 (30.02-48.08)	31.79 (27.84-40.42)	41.22 (31.38-53.57)								

^a^Prior to discharge (baseline).

^b^At 12 weeks following discharge.

^c^At 24 weeks following discharge.

^d^MSES: Moorong Self-Efficacy Scale.

^e^Normally distributed data.

^f^SF-36: 36-item Short-Form Health Survey.

^g^Nonnormally distributed data. After conversion to normally distributed data by Box-Cox transform, the data were subsequently analyzed using repeated measures analysis of variance.

^h^PCS: physical component summary.

^i^MCS: mental component summary.

**Table 5:** Simple effect analysis results of the Moorong Self-Efficacy Scale between the study and control groups.

Outcome	Time	*df*	Mean square	*F*	*P* value
MSES^a^	T_0_^b^	1	961.724	2.400	.12
MSES	T_1_^c^	1	50.000	0.125	.72
MSES	T_2_^d^	1	3409.020	8.506	.004
Social function self-efficacy	T_0_	1	62.082	1.563	.21
Social function self-efficacy	T_1_	1	4.939	0.124	.73
Social function self-efficacy	T_2_	1	345.469	8.698	.003
General self-efficacy	T_0_	1	62.082	1.926	.17
General self-efficacy	T_1_	1	35.520	1.102	.30
General self-efficacy	T_2_	1	205.755	6.382	.01
Person function self-efficacy	T_0_	1	232.663	2.400	.12
Person function self-efficacy	T_1_	1	1.235	0.013	.91
Person function self-efficacy	T_2_	1	648.000	6.684	.01

^a^MSES: Moorong Self-Efficacy Scale.

^b^Prior to discharge (baseline).

^c^At 12 weeks following discharge.

^d^At 24 weeks following discharge.

### SF-36 in the Study and Control Groups

**Figure 6:**
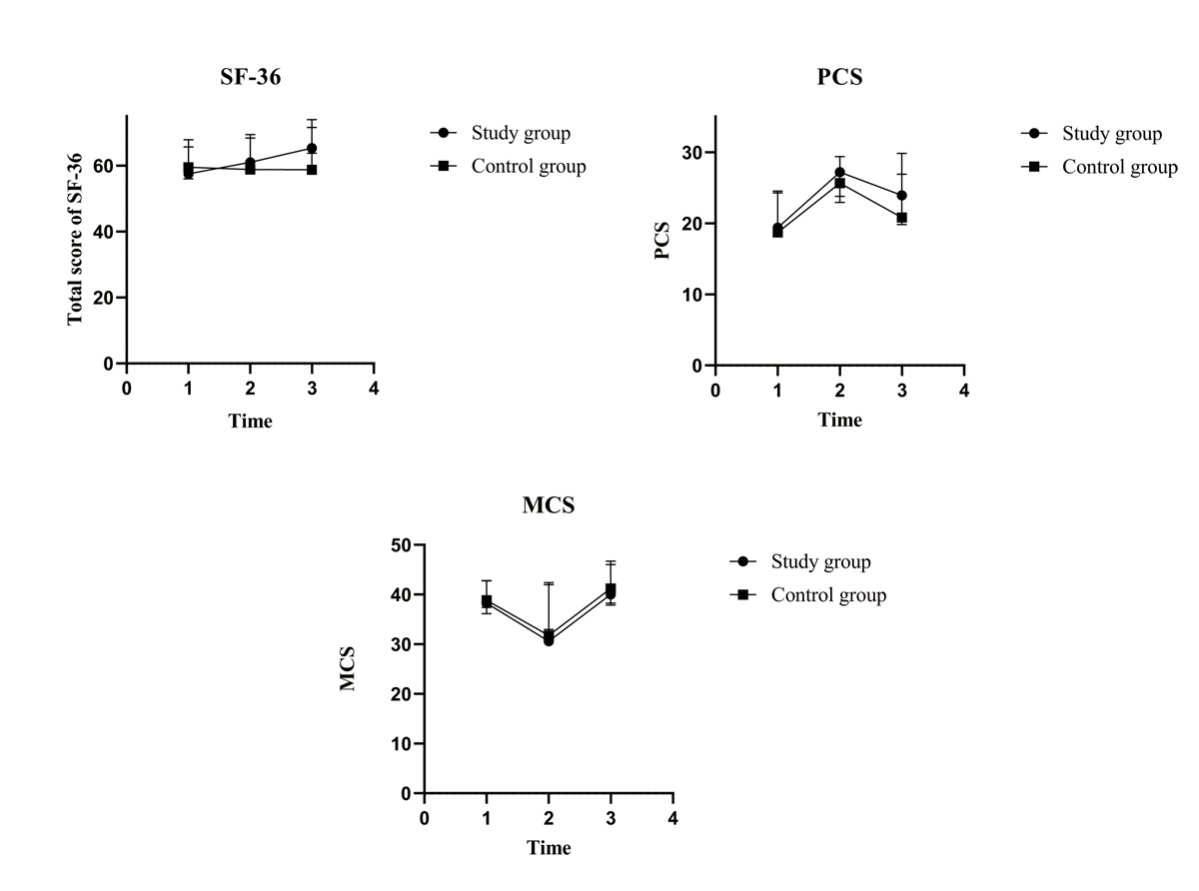
Trend charts of total 36-item Short-Form Health Survey (SF-36) scores and scores of two components. MCS: mental component summary; PCS: physical component summary; time 1: prior to discharge (baseline); time 2: at 12 weeks following discharge; time 3: at 24 weeks following discharge.

The total scores and two-component scores of the SF-36 for the study and control groups at T_0_, T_1_, and T_2_ are summarized in Table 4. Differences in the time effects of the total scores and PCS scores were statistically significant (total scores: *F*_2,95_=6.671, *P*=.002; PCS: *F*_2,95_=24.516, *P*<.001). However, there were no differences between the study and control groups in the time effects in the MCS, interaction effects, and group effects. Trend charts (Figure 6) showed that the total scores in the study group trended upward, whereas the control group scores decreased over time. The PCS scores of both groups showed an increasing tendency at the beginning and then a declining tendency after 12 weeks following discharge. The tendency in MCS scores for both groups was consistent with the opposite tendency in PCS scores.

## Discussion

### Principal Findings

We found that patients in the study group who received app-based transitional care had better levels of self-efficacy than those in the control group. However, there was no significant statistical difference in QOL between the study and control groups.

Our findings indicated that app-based transitional care was effective at enhancing the self-efficacy of patients with SCI. This is consistent with the results of studies involving other populations, such as puerperium women [18], gestational diabetes patients [49], and stoma patients [50]. The study conducted by MacGillivray et al [19] also revealed that a mobile app could enhance the confidence of patients with SCI in bowel self-management. The study showed that reinforcing the health-related knowledge of patients can enhance their self-efficacy and self-management behaviors [51]. In our study, the follow-up indicators were extracted from the ICF components, body function, body structure, activities and participation, and contextual factors, which covered the most important issues faced by home-dwelling patients with SCI. These issues included physiological and psychological functional disorders, complications, activities of daily living, and social participation. According to the assessment results of each indicator, standardized health education was provided to patients with SCI, focusing on their knowledge, attitudes, and practice. The delivery and reinforcement of health information from medical staff to patients with SCI may be the main reason for the promotion of self-efficacy in patients. Moreover, app-based transitional care extends social support from professional medical staff to patients. It was demonstrated that higher levels of social support are linked to higher levels of self-efficacy [52]. Compared with the control group, the social support provided to patients and caregivers in the study group by the multidisciplinary team was more diverse. Additionally, the abilities of caregivers in caring for patients were also assessed, and they received interventions in this study. This approach was also valuable for establishing an effective social support system for the patients.

Although we observed that the study group exhibited a greater improvement in QOL total scores than the control group, there were no significant differences observed between the study and control groups. The results were also consistent with those of the study conducted by Kryger et al [53], which examined the effect of an app on patient QOL. Among other tele-interventions for patients with SCI, Shem et al [54] used FaceTime, a videotelephony app installed on an iPad (Apple Inc), to provide a telemedicine program, and it did not greatly improve the QOL of patients with SCI at 6 months following discharge from the hospital. It is established that the effects of SCI on patients can influence their physiological health, mental health, and social participation in the long term [55]. Therefore, the QOL of patients with SCI was not easily altered. QOL is a multidimensional concept, composed of one’s state of the physical, psychological, and social domains [56]. For the physical aspect, the disabilities manifest with paraplegia or tetraplegia, and they seriously affect the functioning and daily activities of patients. For the psychological aspect, patients with SCI often exhibit negative reactions, such as anxiety and depression due to the accident, disabilities, pain, etc, which greatly affect the subjective feelings of patients regarding life [57]. For social participation, some studies have shown that relevant barriers to social participation exist for patients with SCI owing to environmental constraints, the attitudes of others, and family economic status [58]. Therefore, improving the QOL of patients with SCI requires patients, families, health professionals, and even the whole society to provide long-term integrated support covering all aspects of their physical, mental, and social domains. Notably, the QOL total score and PCS score of the study group showed better trends than those of the control group. Fan [59] conducted an RCT and found that the QOL of patients with SCI at 12 months following discharge improved. A longer observation period may provide more valuable information regarding the effects of app-based transitional care on the QOL of patients with SCI.

### Limitations

The study had some limitations. First, when developing the outcome indicators suitable for SCI follow-up, we did not include patients in our expert panel. In the future, quantitative or qualitative research could be performed to make the follow-up indicators more comprehensive and meet patient needs. Second, the sample size of the study was small. Owing to the low incidence of SCI and the limitation on the disease duration (within 2 years), only 98 eligible patients recruited from four research centers completed the follow-up. Third, the observation period was limited. We followed discharged patients with SCI up to only 24 weeks. Thus, the long-term effects of app-based transitional care should be further investigated.

## Appendix

Multimedia Appendix 1 CONSORT-EHEALTH checklist V1.6.

## Abbreviations

AD: autonomic dysreflexia
CVI: content validity index
ICF: International Classification of Functioning, Disability, and Health
MCS: mental component summary
MSES: Moorong Self-Efficacy Scale
PCS: physical component summary
QOL: quality of life
RCT: randomized controlled trial
SCI: spinal cord injury
SF-36: 36-item Short-Form Health Survey
